# Robust Estimation of Carbon Monoxide Measurements

**DOI:** 10.3390/s20174958

**Published:** 2020-09-02

**Authors:** Wilmar Hernandez, Alfredo Mendez

**Affiliations:** 1 Facultad de Ingeniería y Ciencias Aplicadas, Universidad de Las Américas, Quito 170125, Ecuador; 2 Departamento de Matemática Aplicada a las Tecnologías de la Información y las Comunicaciones, ETS de Ingeniería y Sistemas de Telecomunicación, Universidad Politécnica de Madrid, 28031 Madrid, Spain; alfredo.mendez@upm.es

**Keywords:** carbon monoxide, nonparametric statistical inference, robust central tendency estimation, robust scale estimation, nonparametric confidence interval, robust confidence interval

## Abstract

This paper presents a robust analysis of carbon monoxide (CO) concentration measurements conducted at the Belisario air-quality monitoring station (Quito, Ecuador). For the analysis, the data collected from 1 January 2008 to 31 December 2019 were considered. Additionally, each of the twelve years analyzed was considered as a random variable, and robust location and scale estimators were used to estimate the central tendency and dispersion of the data. Furthermore, classic, nonparametric, bootstrap, and robust confidence intervals were used to group the variables into categories. Then, differences between categories were quantified using confidence intervals and it was shown that the trend of CO concentration at the Belisario station in the last twelve years is downward. The latter was proven with the precision provided by both nonparametric and robust statistical methods. The results of the research work robustly proved that the CO concentration at Belisario station in the last twelve years is not considered a health risk, according to the criteria established by the Quito Air Quality Index.

## 1. Introduction

Carbon monoxide (CO) is a colorless and odorless gas that when found in the air in large concentrations is harmful to both humans and animals. This gas is produced when fossil fuels are burned and, therefore, internal combustion engines and vehicles or machinery whose operating principle is based on burning fossil fuels are among the greatest sources of CO, which is an air pollutant of concern worldwide [1]. Additionally, gasoline-powered pressure washers, propane-powered forklifts, propane-powered resurfacing machines, and gasoline-powered appliances, among others, can cause CO poisoning when are not used correctly in some applications [2]. The most important source of CO is motor vehicle exhaust [3]. However, catalytic convertors have reduced automobile exhaust emissions of CO [4].

In addition, detonation of explosives employed in blasting can produce CO and people living near a blast site can be affected [2]. Furthermore, smoke-polluted environments and hookah smoking are also sources of CO exposure [2].

CO is a silent killer [4] and the type of CO poisoning that a human being can suffer will vary depending on the level of CO concentration to which they are exposed and the length of time that such exposure lasts. For example, a human being who is exposed to high levels of CO concentration for a long time may go into a coma or even die [4]. Moreover, the most common symptoms of CO poisoning include the following: headaches, dizziness, vomiting, nausea, dyspnea, chest pain, tachycardia, blurred vision, confusion, palpitations, dysrhythmias, cardiac arrest, myocardial ischemia, seizures, respiratory arrest, and coma, among others [4,5,6].

The aforementioned justifies the need to robustly analyze the information from CO measurement devices. In this sense, inferential statistical analysis plays a fundamental role, because this type analysis allows to estimate the central tendency and dispersion of the data and to determine confidence intervals for location estimates of the variables under study.

The main objective of this paper is to carry out the robust estimation [7,8,9] of a set of twelve years of CO concentration measurements performed at Belisario air-quality monitoring station in Quito (Ecuador) [10]. The time interval in which these measurements were performed is from 1 January 2008 to 31 December 2019.

Some previous research works in which the statistical analysis of CO concentrations has been carried out are the following; In order to study the quality of air in underground mines, a time series describing CO concentration in a copper ore mine in Poland, from 28 October 2014 to 28 December 2014, was analyzed [11]. To carry out the aforementioned analysis, statistical models were used. Also, several parametric distribution functions were considered, the least squares method was used to estimate parameters, the K-means algorithm was used to classify the CO concentration, and the missing observations were either filled by using interpolation based on adjacent values or the time periods corresponding with missing information were not taken into account.

The kernel principal component analysis was used [12] to extract the nonlinear mixed gas characteristics of different components, and the K-nearest neighbor algorithm was used to recognize the target gas. In [12], a gas identification and concentration detection method was presented and a multivariable relevance vector machine was used to detect the concentration of the hybrid gas. The aforementioned method was validated by using CO and methane (CH_4_).

An example of using probabilistic models for the analysis of air pollution variables can be found in [13]. In short, the Rasch probabilistic model was used in [13] to define a measure of atmospheric pollution, integrating pollutants such as CO, among others, and several climatic factors. The study presented in [13] was carried out in Southwest Spain and, for the analysis, data of pollutants were collected from 1 January 2016 to 31 December 2016. Furthermore, the mean value, standard deviation, and minimum and maximum values were used to assess the proposed probabilistic model.

Another example of statistical analysis of CO concentration in urban cities can be found in [14], where the study was performed in seven locations in Los Angeles Basin (California, USA), from 1955 to 1972. In this case, the statistical analysis of the trend of CO concentrations was carried out by using time series analysis, and the relationship between meteorological variables and CO concentrations was assessed.

Veterans are also affected by CO poisoning and [15] was aimed at describing the distribution and determinant factors of CO poisoning in veterans. In short, in [15] it is said that the U.S. Veterans Health Administration (VHA) provides care to over 9 million veterans and that there is a great need to study in depth the trend of CO poisoning among them. In [15], demographic variables were analyzed and compared to users of VHA care from 2010 to 2017, and the results were supported by 95% confidence intervals. Moreover, in order to test for statistical significance, the two-tailed z test for proportions was used.

Descriptive statistics can also play a key role in the preliminary analysis of CO concentration measurement data, because it can be a quick indicator of trends in deaths from poisoning. For example, in [16] descriptive statistics was used to analyze the trend in deaths due to CO poisoning in Turkey from 2008 to 2017.

Another study of the association between CO poisoning and mortality can be found in [17]. Specifically, short-term associations between CO and daily mortality because of cardiovascular diseases in China, from 2013 to 2015, were analyzed [17]. Additionally, over dispersed generalized linear models were used in [17] to estimate associations between the concentration of CO and daily mortality due to strokes and to cardiovascular and coronary heart diseases. Moreover, in [17] Bayesian hierarchical models were used to obtain national and regional average associations.

Furthermore, the robustness of the effects that CO poisoning has on cardiovascular mortality was evaluated in [17] using fitted two-pollutant models. However, the concept of robustness introduced in [17] was not in the sense of [7,8,9]. Specifically, the authors of [17] said that that the association between CO and mortality was robust if the significance of the predictor variable in a meta-regression model was very little.

An uncertainty analysis of CO measurements performed at the Izaña mountain station (Tenerife, Spain) was developed in [18]. Additionally, time series analysis was used to study the daily nighttime mean of CO concentration and, in order to perform the study, a least-squares fitting to a nonlinear function was carried out. This function consisted of a quadratic year-on-year component plus four Fourier harmonics that represented an annual cycle.

Sometimes observations of CO concentration do not follow a Gaussian distribution. Therefore, these observations cannot be analyzed using classical statistical inference methods. This happens in the present research work. But, it has also happened in research works carried out by other authors. For example, in [19] a statistical analysis of measurements of gas emissions from gasoline-powered vehicles in Irbid Directorate (Jordan), was carried out. In that paper, in order to analyze vehicle emissions of CO and other pollutants, 1000 vehicles were tested. In summary, the study performed in [19] was aimed at determining whether there were significant differences in the mean value of several emissions of pollutants which came from vehicles with different characteristics. With the purpose of conducting the above-mentioned study, nonparametric tests such as the Kruskal-Wallis test and the Mann-Whitney U test [20,21] were used [19]. Other examples of recent publications in which nonparametric tests have been used to analyze measurements of air pollution variables can be found in [22,23,24,25,26,27,28,29].

In the present paper, robust statistics [7,8,9] is used to analyze 12 years of measurement results of CO concentration at Belisario station [10], which is one of the most important stations of Quito Metropolitan Atmospheric Monitoring Network (QMAMN) [30]. QMAMN is part of the Ministry of the Environment of Ecuador and in Quito this network has nine air-quality monitoring stations, which are located in very important parts of the city.

The statistical analysis of different variables of air pollution in Quito was also carried out superficially in [30]. In fact, in [30] a robust analysis of the air pollution variables was not carried out, and the statistical tools used to analyze CO concentration were only the mean and maximum values. Therefore, it is necessary to complete what appears in [30] with a formal and rigorous study of the numerical results of the measurements of CO concentration levels in Quito. In this sense, the research work presented here could serve as a reference material to comprehensively analyze the results of the CO concentration measurements that have been carried out at the Belisario air quality monitoring station, in the last twelve years.

The objectives of this paper are the following:

(1) Construct sets of variables that represent the 12 years of CO concentration measurements under study, the months of the year, and the hours of the day, to determine statistical parameters that establish similarities and differences between the elements of these sets of variables.
(2) Classify CO concentration measurements by using different methods of estimating the central tendency and dispersion of the data. Specifically, classic, nonparametric, resampling, and robust methods are used.
(3) Categorize and discriminate CO concentration measurements using confidence intervals. These confidence intervals are constructed at the 95% confidence level and are of the following types: classic, nonparametric, bootstrap, and robust confidence intervals.
(4) Find periodicities in the sets of variables that represent the repetition of certain behaviors each time a certain time interval elapses.

Previous research papers that have been entirely focused on the use of robust statistics to analyze the behavior of air pollution variables are those shown in [31,32,33]. In addition, other research papers in which robust estimators have been used to analyze air pollution variables are [34,35].

The rest of the paper is organized as follows: Section 2 gives information about the study site and shows summary statistics. Section 3 is devoted to carrying out the data analysis by using nonparametric statistical inference techniques. Section 4 is aimed at performing the robust estimation of the CO concentration measurements. The aim of Section 5 is to perform a discussion of the results. Finally, the conclusions are given in Section 6.

## 2. Study Site and Summary Statistics

The study site was the Belisario station and information about this monitoring station can be found in [10,30]. According to [30], the data were collected using CO analyzers from Thermo Fisher Scientific, model 48i [36], which is a reference-level instrument that serves as a measurement standard in many countries (e.g., it is designated as a Federal Equivalent Method by the US EPA).

In this paper, each data represents a CO concentration value for each hour and said data are the result of the arithmetic mean of the CO concentrations that have been measured every 10 min of the corresponding hour represented by the data [30]. According to [37], in order to calculate the averages, 75% of the valid records were covered.

For the analysis, the data collected from 1 January 2008 to 31 December 2019 was considered, and the results of the analysis carried out refer to most of the data collected. Here, it will be analyzed whether the oscillations of the measurements are due to random variations or they indicate that the measurements are different from each other. The aforementioned will be carried out using nonparametric and robust statistics tools.

Since the data collected begins on 1 January 2008, with a sampling rate of one hour, and refers to a full 12 years, this would mean 105,193 data. However, since some data does not appear, others have negative values and one has an exceptionally high value compared to the rest, the analysis has been carried out with more than 96% of all the data; that is, only less than 4% of the total data has been lost. Negative values were removed because they cannot be valid. Nevertheless, the values equal to zero were taken into account, because these could represent valid measurements that were carried out at certain time instants. On the other hand, there was an excessively large point value that was also removed, because it was clearly seen that it could not be valid and that it also had no relationship with the rest of the values of the data set.

In this research work, there were no data scarcity problems and the time instants corresponding to missing information were not taken into account, because the robust analysis was carried out based on the information that was actually provided by the measurement instrument without need to perform any kind of interpolation, which represents one of the strengths of robust statistical inference.

With the data available, consisting of year, month, day, hour, and amount of CO concentration in milligrams per cubic meter ($\mathrm{mg}/m^{3}$), the analysis and interpretation of these will be carried out with the aim of finding relationships between said data. The variables under analysis are $X_{k}$, k=1,…, 12, which are the CO concentrations in 2008, 2009, and so on until 2019. That is $X_{1}=2008,\;X_{2}=2009,\;\ldots ,\;X_{12}=2019$.

**Figure 1:**
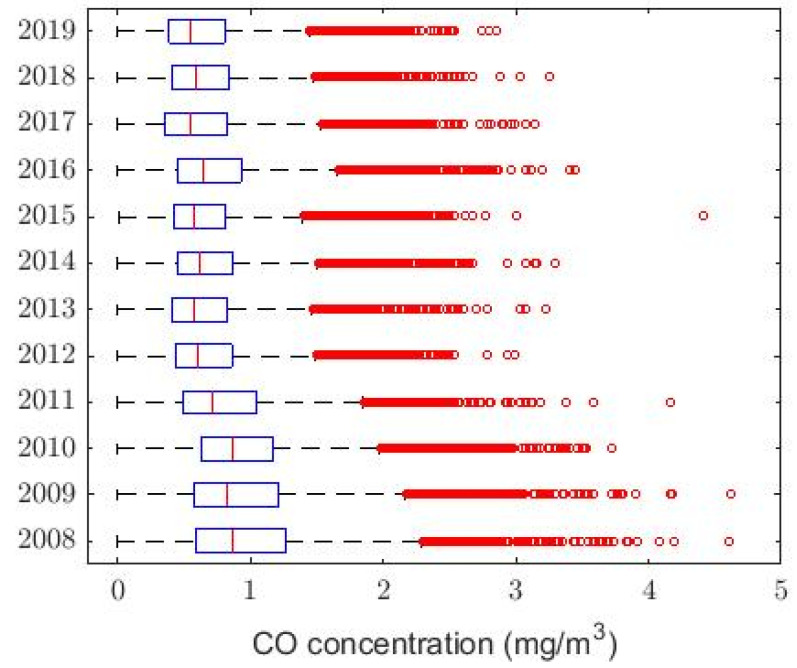
Box plot diagram of all years. The red circles represent the outliers.

**Figure 2:**
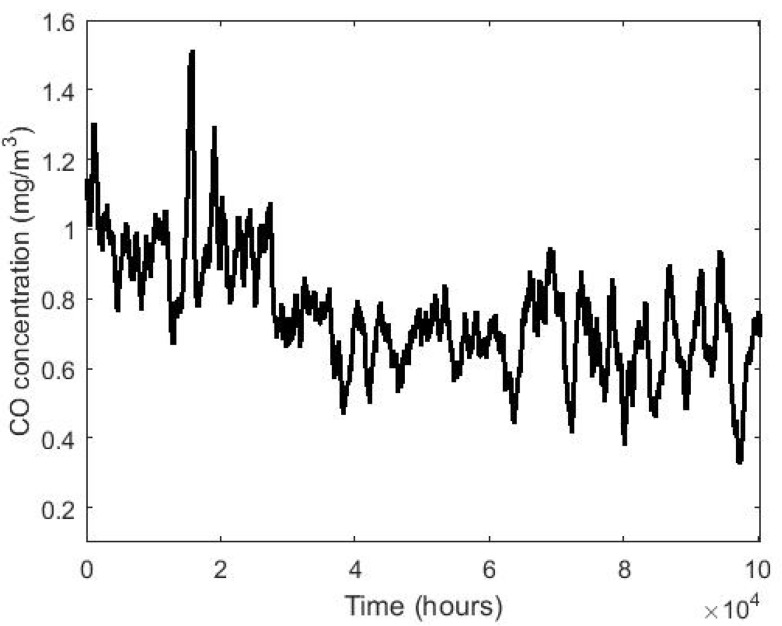
Moving average of the time series consisting of the values of the CO concentration from 1 January 2008 to 31 December 2019.

**Figure 3:**
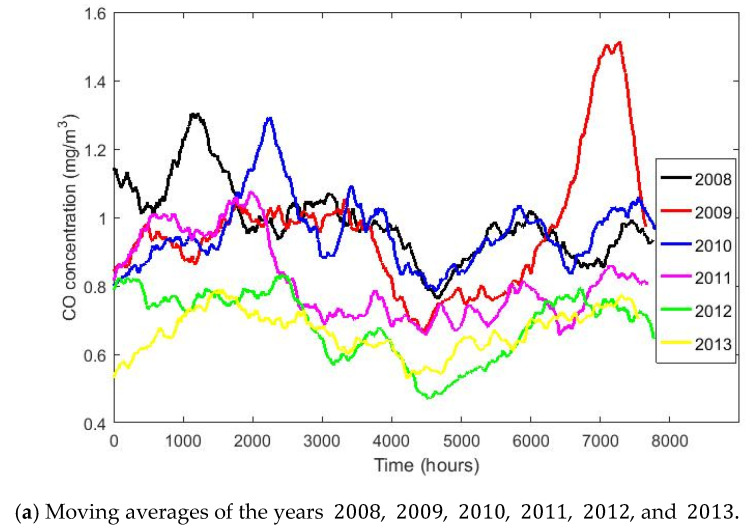

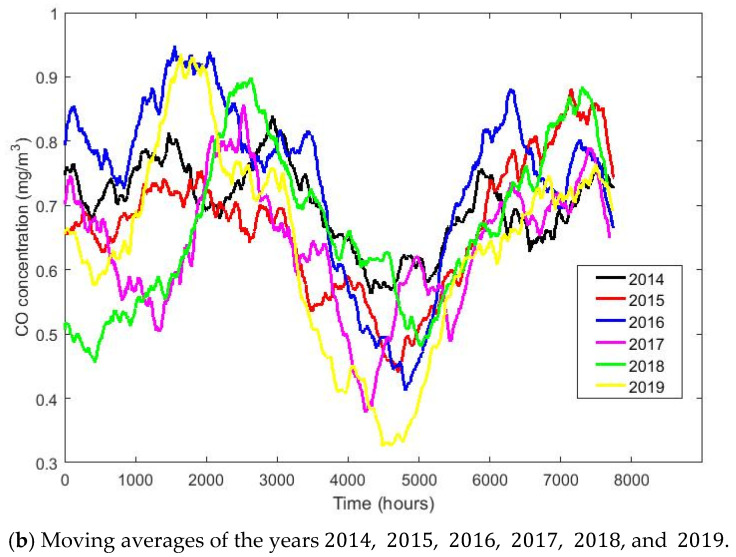
Moving averages of half of the years.

Figure 1 shows the box plot diagram of the variable CO classified by years, and Figure 2 and Figure 3 show three graphs of moving averages (MAs), one graph shows the MA of all the years and two others show the MA of half of the years. This smoothing technique is used in time series studies [38,39] and will be used here to analyze the trends of the variables. Although there are different types of MA smoothing, the simplest will be used in this paper. This type of smoothing by MA consists of the following: Given a value m less than the total number of data, the mean of the data set $x_{h},\;x_{h-1},\;\ldots ,\;x_{h-m+1}$ is found for each h≥m. In this way, each data loses its individual influence, although m−1 observations are lost. In this paper, the MA of size 720 has been considered, since 720 is the number of data that would be in a full 30-day month.

The boxplot and moving average graphs shown in Figure 1, Figure 2 and Figure 3 show that all variables (years) appear to behave similarly to each other except in 2009. In addition, Figure 1 shows that the number of observations that are extremely high, compared to available values, decrease as the years pass. Moreover, Figure 2 indicates a trend to decrease the CO concentration continuously as time passes.

In order to provide information that quickly supplies a simple description of the measurement results, Table 1 shows a statistical summary of the data. From Table 1, it can be seen that for each year there are approximately between 94% and 97% of all possible data. Also, this table shows that for all the variables the mean is higher than the median, that the skewness is positive and that the kurtosis is higher than 5, reaching values higher than 7 in some years.

The aforementioned indicates that it is very likely that all the variables under study come from heavy-tailed distributions [8,40], because, based on the information provided in Table 1, the medians are less than the means, the skewness are greater than zero, the kurtosis they are greater than 3, and it is observed that the values of the standard deviations are not small when compared with the values of the means. Furthermore, from Figure 1 it can be seen that there are many outliers.

This idea is confirmed with the boxplot graph shown in Figure 1, where abnormally high observations are presented every year. Therefore, these observations do not come from Gaussian variables [41]. Furthermore, none of the observations exceeds the desirable level of air pollution that is established by the Quito Air Quality Index (QAQI) [30]. QAQI establishes that the maximum value of the desirable level of air pollution is equal to $5\;\mathrm{mg}/m^{3}$. In any case, CO concentrations below $5\;\mathrm{mg}/m^{3}$ may be considered safe or low risk for human beings. Therefore, for the case under study, it can be said that the CO concentration at Belisario station is not considered a health risk.

Finally, due to the fact that there are many observations for each of the variables, the first thing that was done was to try to carry out the statistical analysis using classical inference techniques. Therefore, attempts were made to implement different variable transformations that allowed the variables under study to fit a normal distribution [41]. In this sense, the following variable transformations were performed: sum of constants, logarithms, operations of taking *n*th roots, and inverse functions, among others. However, the results were not as expected, because it was not possible to adequately fit the data for one year to known random variables that were not heavy tails, and a fundamental characteristic of heavy-tailed distributions is that the central limit theorem does not work for them. Therefore, there was no way to fit any of the variables to a normal distribution. In fact, the settings that at some point seemed visually appropriate had *P*-values [21] less than 0.005. All this justified the use of nonparametric statistics and robust statistics in this research paper.

## 3. Nonparametric Statistical Inference

This section is aimed at knowing whether the samples of the variables came from the same population and had a common median. To do this, a comparison was made between all the variables aimed at testing whether the differences between the medians were due to the variability of measurements or due to random causes. With respect to the aforementioned, the variability of the observations could be produced by particular characteristics of the instants of time in which the measurements were conducted. However, random causes could be produced by weather conditions or noise introduced by measuring instruments, among other things.

In this paper, observations were made on different groups of variables and these variables were considered to be linearly independent, because the linear correlations between the variables were close to zero. In other words, the linear dependence between the variables was not strong. However, it is important to mention that in this research work the existence of nonlinear dependencies between variables was not studied, because this is out of the scope of the paper.

In this paper, in order to study whether the distributions of the variables were the same or not, the Wilcoxon rank sum test [20,21] was used to test whether the data collected in the variables under study comes from distributions with equal medians, as was also done in [22,23,31].

To carry out the hypothesis test, the null hypothesis was considered to be $H_{0}:\;Median=M_{0}$, and the alternative hypothesis was $H_{1}:\;Median\neq \;M_{0}$. Therefore, if the null hypothesis is assumed to be true and also that the quantities observed during all the years are stable, then half of the observations of each year will be less than $M_{0}$ and the rest of the observations will be greater than that amount. Here, the significance level was α=0.05 and the confidence level was (1−α). Lastly, the nonparametric bilateral confidence intervals for the median were calculated as in [31,33].

**Figure 4:**
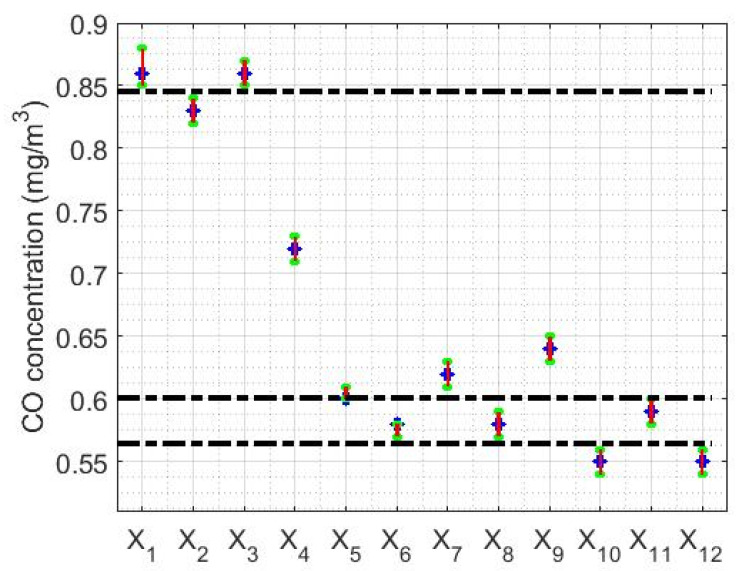
Confidence intervals for the median of each variable. The dashed lines are used to establish the separations between the different categories in which the variables under study are grouped.

The limits of the confidence intervals found in this paper are shown in Table 2, being the confidence level equal to 95%. Furthermore, the graphs of the confidence intervals that were found are shown in Figure 4.

From the information provided in Table 2 and Figure 4, it can be seen again that the amount of CO concentration per year at Belisario station tends to decrease, because as the median decreases the interval shifts to lower values. At this point, it is important to mention that the lengths of the intervals are very small due to the large number of samples available.

In addition, once the Wilcoxon rank sum test was performed, the following was verified:

(1) The medians of the variables $X_{1}$ and $X_{3}$ are homogeneous.
(2) The medians of the variables $X_{6}$, $X_{8}$ and $X_{11}$ are homogeneous.
(3) The medians of the variables $X_{10}$ and $X_{12}$ are homogeneous.
(4) The medians of the variables $X_{2}$, $X_{4}$, $X_{5}$, $X_{7}$ and $X_{9}$ do not coincide with any other.

Therefore, the amount of CO concentration per year can be grouped into four categories, which are indicated in Figure 4, separated by the black horizontal dashed lines. Specifically, the years 2008 ($X_{1}$) and 2010 ($X_{3}$) are in one category, the years 2009 ($X_{2}$), 2011 ($X_{4}$), 2012 ($X_{5}$), 2014 ($X_{7}$) and 2016 ($X_{9}$) are in another category, the years 2013 ($X_{6}$), 2015 ($X_{8}$) and 2018 ($X_{11}$) are in a third category, and the years 2017 ($X_{10}$) and 2019 ($X_{12}$) are in the fourth category.

Before concluding this section, it is important to mention that the fact that the CO concentration has been decreasing over the years could be explained by the environmental policies that have been carried out in the city of Quito in recent years. These results could indicate that these policies, among other things, could be part of the reasons why better results have been obtained.

## 4. Robust Estimation

In this paper, robust methods [7,8,9] were used to carry out the estimation of the central tendency and dispersion of the data in such a way that the results of the analysis were not affected by extreme values [31,32,33].

A useful technique for characterizing robust statistics is the influence curve [42]. This technique aims to measure the influence that an observation has against all other observations. In fact, if the estimators are not robust, then it may happen that the influence curves are not bounded. Therefore, when this happens, the estimator can be greatly affected by an observation that is very far from the rest of the data. With robust estimators, the influence curves are bounded and the estimators are practically insensitive to observations that deviate from the data set.

In this paper, robust estimators were applied to sample order statistics [21]. In short, the ordered sample of $X_{1},\ldots ,\;X_{n}$ is given by $X_{\left(1\right)}\leq X_{\left(2\right)}\leq \ldots \leq X_{\left(n\right)}$, where the observations with the lowest value and the highest value are $X_{\left(1\right)}$ and $X_{\left(n\right)}$, respectively.

### 4.1. Central Tendency Estimators

According to [7,8,9], the location statistics are used to indicate around which value most of the data, with which it is intended to obtain deductions, are grouped to determine the center of the distributions. In this paper, in addition to the mean and median, other statistics will be used.

The *L*-location estimators used in this paper were the following:

(1) Trimean (TM) [7,43].
(2) α-trimmed mean (T(α)) [7,8,9]
(3) α-winsorized mean W(α) [7].

Also, the *M*-location estimators [7,8,9] used in this paper were the following:

(1) Andrew’s wave ($T_{wa}\left(c\right)$) [7,9].
(2) Biweight ($T_{bi}\left(c\right)$) [7,8].

**Figure 5:**
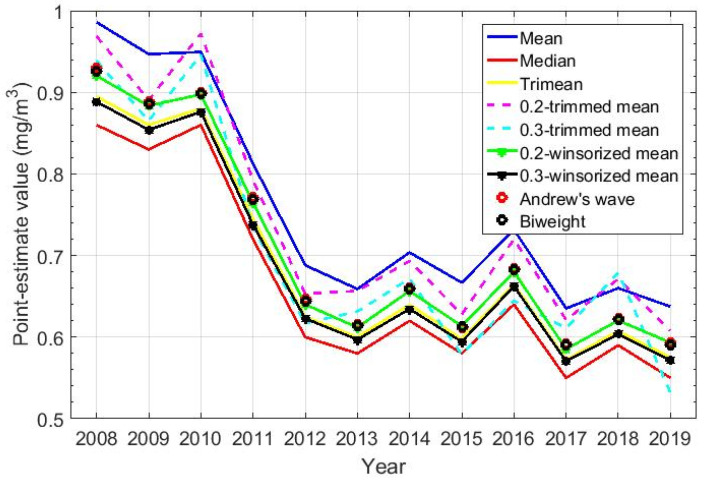
Graphical representation of the location estimates for the twelve years under study. Location estimators: mean, median, trimean, 0.2-trimmed mean, 0.3-trimmed mean, 0.2-winsorized mean, 0.3-winsorized mean, Andrew’s wave, and biweight.

The estimates of the above-mentioned statistics are shown in Table 3. In addition, this table also shows the following estimates: 0.2-trimmed mean, 0.3-trimmed mean, 0.2-winsorized mean, and 0.3-winsorized mean. Furthermore, Figure 5 shows classic and robust statistics of the variables, which correspond to those shown in Table 3. Figure 5 shows that there is a pronounced decrease from 2008 to 2012 and, from 2012 onwards, a stabilization is observed in all the estimates found, with a slight decrease. Note that, in general, all measures of centrality for each year fluctuate between the median and the mean.

### 4.2. Scale Estimators

In this paper, the variability of the data is going to be formalized through scale estimators. In accordance with [8], any statistic satisfying both the shift invariance condition and the scale equivariance condition is a dispersion estimate. The scale estimators that will be used in this paper are the following:

(1) Sample standard deviation ($s_{x}$) [7,8].
(2) Mean absolute deviation ($MAD_{mean})$ [7,8].
(3) Median absolute deviation (MAD) [7,8].
(4) One-half of the fourth-spread (SRH) [7,44].
(5) Least median squares (LMS) [8].
(6) Winsorized standard error ($s^{W}\left(\alpha \right)$) [9].
(7) Andrew’s wave ($s_{\omega a}\left(c\right)$) [7].
(8) Biweight ($S_{bi}\left(c\right)$) [7,8].
(9) Estimator based on a subrange ($C_{n}^{\alpha }$) [45].

**Figure 6:**
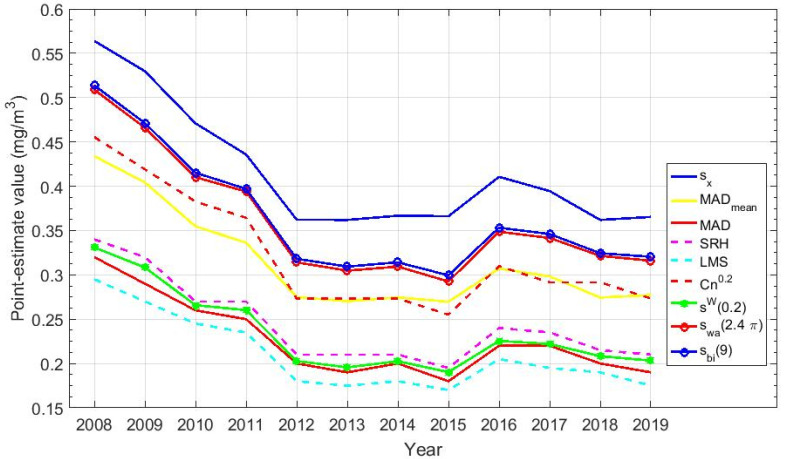
Graphical representation of the scale estimates for the twelve years under study. Scale estimators: sample standard deviation $(S_{x}$), mean absolute deviation ($MAD_{mean})$, median absolute deviation (MAD), one-half of the fourth-spread (SRH), least median squares (LMS), estimator based on a subrange ($C_{n}^{\alpha }$), winsorized standard error ($s^{W}\left(0.2\right)$), Andrew’s wave ($s_{\omega a}\left(2.4\pi \right)$), and biweight ($S_{bi}\left(c\right)$).

The point estimates of scale are shown in Table 4. Furthermore, Figure 6 shows the graphical representation of the point estimates of scale of the variables, which correspond to those included in Table 4.

In Figure 6, it can be seen that all the estimates are upper bounded by the standard deviation and lower bounded by the point estimator least median of squares. In addition, it is observed that the estimators of scale based on the Andrew’s wave and the biweight are very similar to each other, as was the case with the estimators of location based on the Andrew’s wave and the biweight. Moreover, there is a slight drop in the value of all the estimates from 2008 to 2012 and then they stabilize, which could indicate that the increase in the amount of CO concentration produced an increase in its variability, since that the lower limit is always zero.

### 4.3. Confidence Intervals

In this section, following the methodology used in [32] and suggested in [7,8], the confidence intervals were established with a location parameter, a scale parameter, and a constant related to the Student’s t distribution. Furthermore, said constant was selected following the indications given in [46,47]. In what follows, $t_{\nu ,q}$ means the *q*-th quantile of the Student’s t distribution with ν degrees of freedom (DOF). In this paper, the estimators shown in Section 4.1 and Section 4.2 were used to build confidence intervals. The pair of estimators were as follows [32,33]:

(1) $\left(\bar{X},s_{x}\right)$, where $\bar{X}$ is the mean.
(2) $\left(M_{e},\;MAD\right)$, where $M_{e}$ is the median.
(3) $\left(M_{e},\;IQR\right)$, where IQR is the interquartile range.
(4) $\left(T\left(\alpha \right),s^{W}\left(\alpha \right)\right)$.
(5) $\left(T_{wa}\left(c\right),s_{wa}\left(c\right)\right)$.
(6) $\left(T_{bi}\left(c\right),s_{bi}\left(c\right)\right)$.

**Figure 7:**
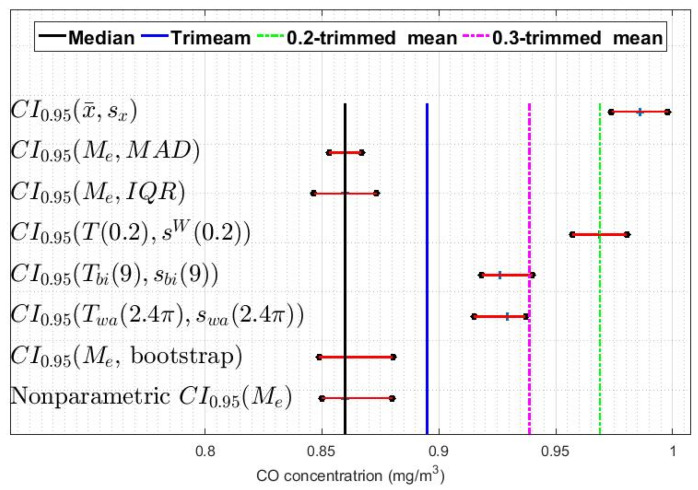
95% confidence intervals ($$CI_{0.95}$$) for $X_{1}\;\left(2008\right)$: classic, nonparametric, bootstrap, and robust confidence intervals.

**Figure 8:**
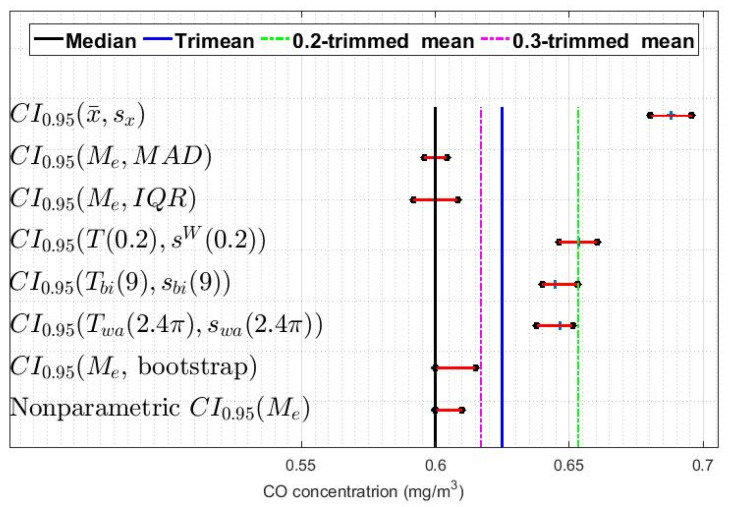
95% confidence intervals ($CI_{0.95}$) for $X_{5}\;\left(2012\right)$: classic, nonparametric, bootstrap, and robust confidence intervals.

**Figure 9:**
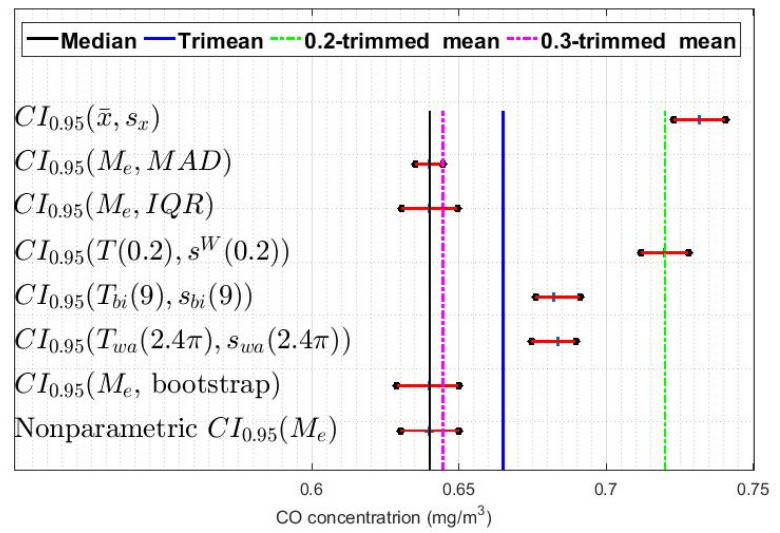
95% confidence intervals ($CI_{0.95}$) for $X_{9}\;\left(2016\right)$: classic, nonparametric, bootstrap, and robust confidence intervals.

In addition, confidence intervals based on a bootstrap method were built [9,32,33]. With all of the above, eight confidence intervals were constructed for each of the twelve variables: one classic, one nonparametric, one using the bootstrap method, and five robust. In Figure 7, Figure 8 and Figure 9, these intervals are shown for three of the twelve variables that have been analyzed, specifically those corresponding to the leap years included in the study. Showing more figures would not provide relevant information.

Figure 7, Figure 8 and Figure 9 show that, in general terms, the variables present similar characteristics regarding the confidence intervals. For example, it can be seen that the classic confidence intervals are the ones that are pushed furthest towards high values, while the median-based confidence intervals are those that are shifted towards the lowest values. Note that this result is consistent with what was said in Section 2, in that it is very likely that the distributions of the variables are heavy-tailed distributions.

Furthermore, Figure 7, Figure 8 and Figure 9 show that among the median-based confidence intervals, the nonparametric intervals and the bootstrap-based intervals are very similar. Also, these figures reflect that the intervals based on the median and the median absolute deviation are the narrowest.

With respect to the confidence intervals based on Andrew’s wave and biweight, it can be said that these have similar characteristics in all the variables and that they are located between values that are to the right of the intervals based on the median and to the left of the intervals based on 0.2-trimmed mean.

Finally, Figure 7, Figure 8 and Figure 9 also show that in all the variables the intervals based on the 0.2-trimmed mean location estimators are the second most displaced towards high values, being these intervals those that are closest to the classic intervals.

Due to all the above, the confidence intervals based on the estimators $\left(T\left(\alpha \right),s^{W}\left(\alpha \right)\right)$ and $\left(T_{bi},s_{bi}\right)$ were used to compare the given variables. The reasons for this decision are as follows: first, the classic intervals are unfounded because the underlying distribution is assumed to be approximately normal, which is not true; second, the results obtained with bootstrap estimators and with the point estimators $\left(M_{e},\;MAD\right)$ and $\left(M_{e},\;IQR\right)$ are analogous to the results obtained with the nonparametric estimators seen in Section 3; and, third, the results obtained with the estimators based on the Andrew’s wave and on the biweight are similar, so either of the two estimators could have been chosen.

Table 5, similar to Table 2, includes the limits of the confidence intervals, with a confidence level of 95%, and their length for the estimators $\left(T\left(\alpha \right),s^{W}\left(\alpha \right)\right)$ and $\left(T_{bi}\left(c\right),s_{bi}\left(c\right)\right)$, for α=0.2 and c=9.

**Figure 10:**
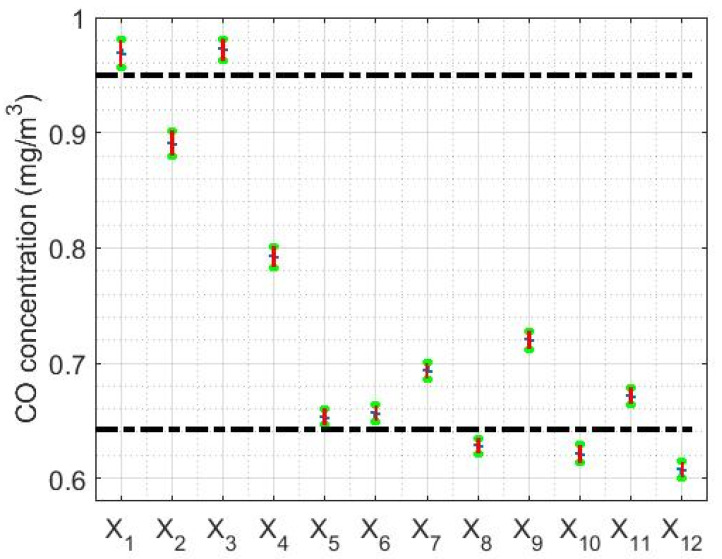
$\left(T\left(0.2\right),s^{W}\left(0.2\right)\right)$ 95% confidence intervals: $X_{1}\;\left(2008\right)$, $X_{2}\;\left(2009\right)$, $X_{3}\;\left(2010\right)$, $X_{4}\;\left(2011\right)$, $X_{5}\;\left(2012\right)$, $X_{6}\;\left(2013\right)$, $X_{7}\;\left(2014\right)$, $X_{8}\;\left(2015\right)$, $X_{9}\;\left(2016\right)$, $X_{10}\;\left(2017\right)$, $X_{11}\;\left(2018\right)$, and $X_{12}\;\left(2019\right)$.

**Figure 11:**
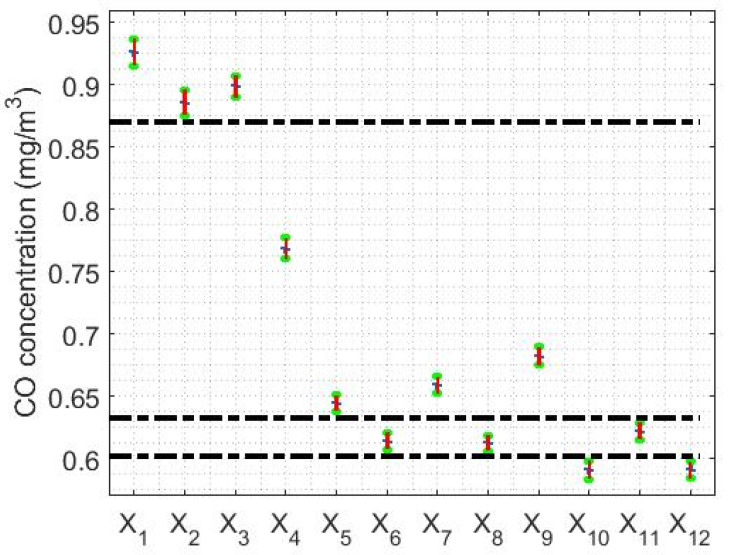
$\left(T_{bi}\left(9\right),s_{bi}\left(9\right)\right)$ 95% confidence intervals: $X_{1}\;\left(2008\right)$, $X_{2}\;\left(2009\right)$, $X_{3}\;\left(2010\right)$, $X_{4}\;\left(2011\right)$, $X_{5}\;\left(2012\right)$, $X_{6}\;\left(2013\right)$, $X_{7}\;\left(2014\right)$, $X_{8}\;\left(2015\right)$, $X_{9}\;\left(2016\right)$, $X_{10}\;\left(2017\right)$, $X_{11}\;\left(2018\right)$, and $X_{12}\;\left(2019\right)$.

The above-mentioned confidence intervals are shown in Figure 10 and Figure 11. In addition, lines have been included in this figure to try to classify the variables, analogous to the classification provided by the Wilcoxon rank sum test for the medians in Section 3. With the biweight estimators, the classification of the variables is similar to that obtained with nonparametric estimators, the only difference is that variable $X_{2}$ is grouped with variables $X_{1}$ and $X_{3}$.

The first observation that is made is that between 2008 and 2012 the tendency to lower CO concentration values is notable, and that from 2012 to 2019 there are fluctuations with a slight downward trend. Regarding the amplitudes, it can be concluded that the confidence intervals found with biweight estimators are narrower than the confidence intervals found with α-trimmed mean and Winsorized standard deviation.

### 4.4. Additional Confidence Intervals

In view of the results found in Section 4.3, it was decided to analyze the same data but with two different groupings. Specifically, variables $Y_{1},\;\ldots ,\;Y_{12}$ have been defined as the amount of CO concentration in each of the months of the year, with $Y_{1}$ being the amount of CO concentration in the month of January of all years, $Y_{2}$ the amount of CO concentration in the month of February of all years, and so on. On the other hand, the variables $Z_{1},\;\ldots ,\;Z_{12}$ have also been defined as the amount of CO concentration grouped from two hours to two hours every day of the year, with $Z_{1}$ being the amount of CO concentration at 0:00 h and at 1:00 h, $Z_{2}$ the amount of CO concentration at 2:00 h and at 3:00 h, and so on.

**Figure 12:**
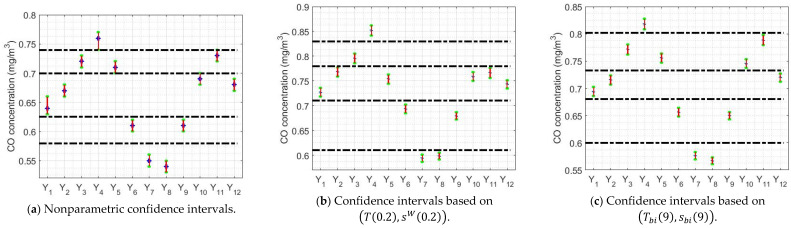
95% confidence intervals for the months: $Y_{1}$ (January), $Y_{2}$ (February), $Y_{3}$ (March), $Y_{4}$ (April), $Y_{5}$ (May), $Y_{6}$ (June), $Y_{7}$ (July), $Y_{8}$ (August), $Y_{9}$ (September), $Y_{10}$ (October), $Y_{11}$ (November), and $Y_{12}$ (December).

**Figure 13:**
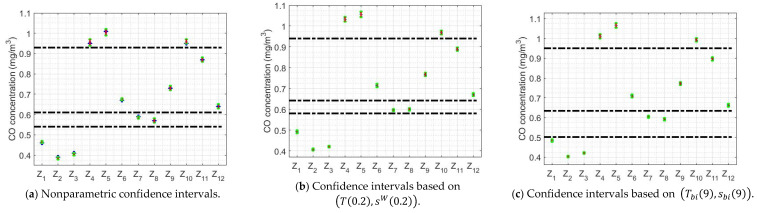
95% confidence intervals for the groups of every two hours of the day: $Z_{1}\;\left(0:00–1:00\right)$, $Z_{2}\;\left(2:00–3:00\right)$, $Z_{3}\;\left(4:00–5:00\right)$, $Z_{4}\;\left(6:00–7:00\right)$, $Z_{5}\;\left(8:00–9:00\right)$, $Z_{6}\;\left(10:00–11:00\right)$, $Z_{7}\;\left(12:00–13:00\right)$, $Z_{8}\;\left(14:00–15:00\right)$, $Z_{9}\;\left(16:00–17:00\right)$, $Z_{10}\;\left(18:00–19:00\right)$, $Z_{11}\;\left(20:00–21:00\right)$, and $Z_{12}\;\left(22:00–23:00\right)$.

The confidence intervals found for the variables $Y_{1},\;\ldots ,\;Y_{12}$ and $Z_{1},\;\ldots ,\;Z_{12}$ are shown in Figure 12 and Figure 13. Regarding these figures, some comments can be made. For example, Figure 12a shows that the highest values are reached in April, followed by a second step in March, May and November. In addition, the variables corresponding to the months of January, February, October and December also behave similarly, with lower values than those previously mentioned. Furthermore, the reduction in CO concentration is very appreciable in June and September. Moreover, the CO concentration values in the central summer months, that is, in July and August, are the lowest of the year. Finally, with respect to the amplitudes of the confidence intervals corresponding to these last two months, it can be said that these intervals appear to be, in general, narrower than the rest of the confidence intervals.

The aforementioned for Figure 12a can be applied quite well to Figure 12b,c, with small differences due to the fact that different estimators were used.

With respect to Figure 13, this figure shows that the only medians that are the same are those of the variables $Z_{4}$, $Z_{5}$ and $Z_{10}$, on the one hand, and those of $Z_{7}$ and $Z_{8}$, on the other hand. part. The rest of the variables can be assumed different from each other and from all the others. In addition, it can be seen that the hours with the lowest CO concentration are those that correspond to the time interval that begins at 0:00 h and ends at 5:00 h. Also, the highest CO concentration values occur between 6:00 h and 9:00 h, and between 18:00 h and 19:00 h. For the rest of the hours of the day, a decrease in the concentration of CO appears in the time interval that goes from 9:00 h to 15:00 h, time of day at which the CO concentration increases again. Finally, the CO concentration begins to decrease from 21:00 h until the next morning.

The aforementioned suggests the existence of a periodic behavior, which also seems to occur when studying the behavior of CO concentration for the months of the year (see Figure 12). However, this certain periodicity in the data did not emerge when these data were analyzed for years.

## 5. Discussion

From an initial statistical summary, it was observed that the values of the CO concentration at the Belisario station are values that are at a desirable level of air pollution, according to the criteria established in QAQI [30]. In addition, it was observed that all the variables present many extreme observations, where said observations are on the right, that is, for high CO concentration values. Furthermore, it was also observed that all the variables present characteristics that are compatible with the possibility that they come from heavy-tailed distributions. Specifically, the variables present medians that are clearly lower than the means, the skewness is greater than zero, the kurtosis is greater than three, and the value of the standard deviation is not small compared to the value of the mean.

Subsequently, a smoothing of the data was performed to decrease the individual influence of each of the data in particular and to highlight possible trends in the data set. This smoothing was performed in the sequence formed by the data corresponding to all the years and for sequences formed for each of the years in particular. All this brought to light that there is a tendency for the values of CO concentration to decrease as the years go by. Likewise, it was also observed that the lowest values are reached in the third quarter of the year and that the highest values occur in the second and fourth quarters of the year. These results are in agreement with the general comments made in [28] about the CO concentration in Quito.

Once the smoothing of the data was performed, an attempt was made to fit all the variables to parametric distributions, through different transformations. This was done with the aim of being able to carry out a statistical analysis applying classical inference techniques, since many observations are available for each variable. However, adequate results were not obtained.

Therefore, due to the impossibility of using classical inference, the study had to be carried out using hypothesis testing and both nonparametric confidence intervals and robust confidence intervals.

This type of exhaustive preliminary analysis, with respect to CO concentration data, is not very frequent, because in general the authors tend to assume independence between the variables, to eliminate outliers and to approximate the remaining data with known parametric distributions. For example, in [11] independence between observations was assumed, peaks in signal amplitude were detected, empirical tails of these peaks were calculated, and theoretical tails of known distributions were fitted to the empirical tails. All of this was done in [11] using classical methods. In [12], kernel principal component analysis was applied to raw data to extract non-linear characteristics from it, and then the K-nearest neighbor algorithm was used for recognition tasks.

On the other hand, in [13] statistical summaries of the data were shown, where the measures of central tendency and dispersion of the data were the following: the mean, the standard deviation, the minimum value, and the maximum value. Then, it focused on the use of the Rach model to define a coherent variable and the interrelation between variables. However, in [15] a statistical summary of the data was not shown, but the analysis was performed using classic confidence intervals at the 95% confidence level and the analysis used the two-tailed Z test for proportions. Additionally, in [16] the preliminary analysis of the data was not shown either, but the descriptive analysis of the data was done in terms of frequency and percentage.

Nevertheless, in [17] the statistical summary of the data was shown, where the measures of central tendency and dispersion used were the mean, standard deviation, range, median, and interquartile range. In addition, for the analysis, the posterior mean and the 95% posterior interval were included. Moreover, Bayesian hierarchical models were used to obtain national-average associations.

In [18], although an initial summary data statistic was not shown, it did explain in detail, exhaustively, the methodology used to discard the data that were not significant for the type of analysis of uncertainty of CO concentration that was performed in that paper. However, the statistical analysis presented in [19] did include statistical summaries of the data, where the authors relied on the mean and standard deviation. Furthermore, in [19] the authors demonstrated that the distribution of the raw data was not normal. Therefore, with the latter, they justified the use of nonparametric statistical methods to carry out the analysis of the CO concentration and other air pollutants.

Regarding the type of nonparametric analysis that was carried out in this research, it can be said that the Wilcoxon rank sum test was used here to compare the medians of the distributions of the variables under study, basing this test on the statistics of the order of samples and on the sign test. Another added value of the present study is that with the nonparametric confidence intervals constructed, the variables could be grouped into different categories, establishing similarities and differences between the data.

Once the nonparametric analysis was carried out, the categorization and discrimination of the data was conducted robustly, because this provides a more in-depth analysis of the characteristics of the CO concentration at the place where the measurements were performed. Here, for the analysis, different robust location and scale statistics were found and, some of them, were used to determine robust confidence intervals. Specifically, the following point estimators were used: the mean, the median, and the trimean. In addition, families of α-trimmed mean estimators, α-winsorized mean estimators, Andrews wave-based estimators, and biweight-based estimators were used, which are defined by the proportion of values not taken into account for the estimation. Here, it was observed that for all the years the point estimates of location were practically limited between the mean and the median. In addition, it was observed that the amount of CO concentration, although all its values were in the range of desirable values according to QAQI, decreased markedly between 2008 and 2012, and that from 2012 onwards the decrease in CO concentration was, in general, much lighter but with year-on-year rises and falls.

On the other hand, the point estimators of scale that were used were the following: the standard deviation, the mean absolute deviation, the median absolute deviation, the one-half of the fourth-spread, and the least median squares. Likewise, regarding the families of scale estimators, biweight midvariance estimators, estimators based on subranges, estimators based on the Andrew’s wave, and estimators based on the Winsorized standard deviation were used. For the estimator families, values that are mentioned in the specialized literature on the subject as suitable values were chosen. The graphical representation of the scale estimators showed that these were bounded inferiorly by the least median squares and superiorly by the standard deviation. Additionally, there is a decrease from 2008 to 2012 and a stabilization from that year until 2019. The decrease is due to the decrease in the number of extreme observations and their value.

The exhaustive robust analysis that has been carried out here on the CO concentration constitutes another added value of the study. Specifically, the technical report presented in [30] does not make an in-depth statistical analysis of the variables of air pollution in Quito. In fact, in [30] only the mean and maximum values are used to analyze the behavior of the CO concentration. Therefore, the research carried out here can be used as reference material to explain how the behavior of the CO concentration in Quito has been from 1 January 2008 to 31 December 2019.

Similar research papers in which robust analysis of other air pollution variables has been performed are those shown in [31,32,33]. The results obtained in this paper are in agreement with those obtained in [31,32,33]. Examples of other research papers that are further from the topic discussed here, but that also have employed some of the robust analysis tools used in this paper are [34,35]. In all cases, the importance of the use of robust methods in the analysis of air pollution variables was highlighted.

The robust bilateral confidence intervals were found using six pairs of robust estimators, three with point estimates and three others with families of estimators from which particular values were selected. In addition, bootstrap confidence intervals were found. Here, it was observed that the confidence intervals at 95% more displaced towards higher values were the classic intervals, because they have their center in the mean, while the confidence intervals more displaced to the left were those that have their center in the median. Likewise, among the confidence intervals centered on the median, the nonparametric intervals and those found by the bootstrap method were wider than those found with robust estimators.

The confidence intervals based on the Andrew’s wave and the biweight were very similar, because the location and scale estimators found in these families were also very similar. On the other hand, the confidence intervals based on α-trimmed mean location estimators, which have the Winsorized variance as variance, produced intervals between the biweight intervals and the intervals based on the Andrew’s wave, on the left, and the classic intervals, on the right.

Due to all the above, the confidence intervals based on the estimators $\left(T\left(\alpha \right),s^{W}\left(\alpha \right)\right)$ and $\left(T_{bi},s_{bi}\right)$ were used to compare the given variables. Again, when the variables were compared using confidence intervals, a downward trend in the CO concentration was observed between 2008 and 2012. Moreover, from 2012 onwards, fluctuations were observed with a slight tendency to decreasing the CO concentration values. Overall, the biweight-based confidence intervals were somewhat narrower than those found with α-trimmed mean. Furthermore, the classifications of the variables found with biweight were similar to those found with nonparametric estimators, the difference was that the variable $X_{2}\;\left(2009\right)$ was added to the category formed by $X_{1}\;\left(2008\right)$ and $X_{3}\;\left(2010\right)$.

To complete the study, the proposed robust confidence interval analysis technique was also applied to clusters consisting of CO concentration measurements of months and clusters of CO concentration measurements in groups of two hours. Here, the variables were classified and it was noted that there was a certain periodicity in both the months and the hours of the day. In this sense, it is observed that the lowest confidence intervals corresponding to the analysis of the months are in the third quarter. Moreover, with respect to the hours of the day, it is observed that there is a certain periodicity, showing minimums in the early morning hours and maximums in the early hours of the working day and in the early hours of the night.

An additional contribution of this study is that the observed periodicities have been shown in terms of robust confidence intervals at the 95% confidence level, categorizing the range of values of the possible periodic wave and measuring differences between categories with the measurement precision provided by robust statistical methods. In addition, it is important to mention that these periodicities are not fixed, but are subject to seasonal variations and even to the character of the day in particular. For example, when considering the CO concentration between 2:00 and 3:00, which is where the lowest CO concentration of the day occurs, said concentration will be different if it is measured in different months. Specifically, the amplitude of the possible periodic signal is not the same if it is measured in April, where the CO concentration is higher, as if it is measured in August, where the concentration is lower.

It is possible that the aforementioned variations in amplitude are due to the time periods in which different activities are carried out in the city. Therefore, the highest concentration of CO when analyzed for the hours is not the same if the day is a holiday or a working day. Furthermore, this means that the signal frequency is not fixed, but is also modulated.

Before concluding this section, it is important to mention that the in-depth analysis of the possible periodic waveform that the CO concentration could have, both for the months and for the hours of the day, has not been included. Therefore, this is a task that remains pending to be carried out in future research work.

## 6. Conclusions

This paper was aimed at performing the robust statistical analysis of CO concentration measurements taken at the Belisario air quality monitoring station (Quito, Ecuador) from 1 January 2008 to 31 December 2019. This is the first time that this type of analysis has been carried out at this monitoring station and its results show that said concentration tends to decrease year after year. Therefore, the measures that the city authorities have been taking in the last twelve years are giving satisfactory results.

The analysis carried out in [30] is an analysis focused on general environmental issues in the city of Quito, which could be strengthened by the in-depth study carried out in this paper. This highlights some of the possible uses of the results obtained in this research work. In this sense, it is important to highlight that in this paper the measurements were classified according to the criteria established by the Quito Air Quality Index to classify air pollution. Additionally, sets of variables were constructed, the variables were categorized, and similarities and differences were also established between the variables. All of this was performed with the precision provided by both nonparametric and robust statistical methods. In this sense, the robust analysis methodology of the CO concentration developed in this paper presents an exhaustive way of carrying out the analysis of measurements of this air pollution variable. Furthermore, one of the advantages of this methodology is that it does not require a large amount of data to carry out the analysis, as has already been demonstrated in [31,32].

In [30], it is mentioned that the main sources of air pollution in Quito are the means of vehicular transportation, which is aggravated by large traffic jams and all the industries that use bunker and fuel oil, highlighting thermo-electric power plants. Moreover, in [30] it is also mentioned that Quito is a narrow and long city, whose central part is located on the slopes of the Pichincha volcano and all travelers who have to travel from one side of the city to the other have to pass through the center of the city, generating traffic jams and consequent air pollution. On the other hand, volcanic eruptions are also sources of air pollution.

What has been said in the previous paragraph shows that, although the exhaustive robust analysis carried out in this paper showed that air pollution due to CO has been decreasing in recent years, it is necessary to improve the urban dynamics of the city. For example, it is proposed that the city comply with quality standards designed specifically for each of its most critical points, in terms of air pollution. In addition, although the quality of the means of transport in Quito have improved significantly, it is proposed to look for more efficient and less polluting means. Likewise, it is proposed to design elements that protect citizens from air pollution while walking on the sidewalks, build more urban parks as air pollution filters and keep citizens informed at all times about the level of air pollution in the city, both in the region through which they travel every day and in the area where they live. All this is in total agreement with what was said in the research work presented in [31].

Finally, based on the time intervals chosen to perform the analysis and represent the results of the research, it was observed that there is a certain periodicity in the CO concentration, both for the months and for the hours of the day. Nevertheless, this periodicity does not occur when the analysis is carried out for the twelve years under study. Therefore, this implies that modeling the possible periodicity of this type of signals is a very complex research topic, where behavior patterns that vary in amplitude, duration and instants of time in which they appear come to light. Trying to model this type of behavior of the CO concentration using mathematical tools is part of a future research work of the authors.

## Figures and Tables

**Table 1:** Summary statistics of the CO concentration measurements.

Year	Count	Mean $\left(mg/m^{3}\right)$	Median $\left(mg/m^{3}\right)$	Standard Deviation $\left(mg/m^{3}\right)$	Skewness	Kurtosis	Minimum $\left(mg/m^{3}\right)$	Maximum $\left(mg/m^{3}\right)$
$2008\;\left(X_{1}\right)$	8483	0.9859	0.8600	0.5640	1.2331	5.0445	0	4.6000
$2009\;\left(X_{2}\right)$	8373	0.9468	0.8300	0.5297	1.3394	5.6238	0	4.6200
$2010\;\left(X_{3}\right)$	8500	0.9494	0.8600	0.4709	1.3400	5.8497	0	3.7200
$2011\;\left(X_{4}\right)$	8398	0.8126	0.7200	0.4358	1.2553	5.4553	0	4.1700
$2012\;\left(X_{5}\right)$	8485	0.6881	0.6000	0.3622	1.3782	5.6295	0	2.9900
$2013\;\left(X_{6}\right)$	8266	0.6589	0.5800	0.3620	1.5447	7.0157	0	3.2300
$2014\;\left(X_{7}\right)$	8477	0.7038	0.6200	0.3668	1.5500	6.7817	0	3.3000
$2015\;\left(X_{8}\right)$	8467	0.6667	0.5800	0.3661	1.6497	7.4224	0.0100	4.4200
$2016\;\left(X_{9}\right)$	8462	0.7317	0.6400	0.4106	1.4815	6.5752	0	3.4500
$2017\;\left(X_{10}\right)$	8408	0.6352	0.5500	0.3945	1.4058	6.0686	0	3.1400
$2018\;\left(X_{11}\right)$	8393	0.6600	0.5900	0.3620	1.3027	5.8100	0	3.2500
$2019\;\left(X_{12}\right)$	8457	0.6374	0.5500	0.3654	1.3281	5.4734	0	2.8500
Total	101,169	0.7566	0.6500	0.4401	1.5245	6.6785	0	4.6200

**Table 2:** Confidence interval limits for the median of each variable α=0.05.

Variable	Lower Limit $\left(mg/m^{3}\right)$	Upper Limit $\left(mg/m^{3}\right)$
$X_{1}\;\left(2008\right)$	0.85	0.88
$X_{1}\;\left(2009\right)$	0.82	0.84
$X_{3}\;\left(2010\right)$	0.85	0.87
$X_{4}\;\left(2011\right)$	0.71	0.73
$X_{5}\;\left(2012\right)$	0.60	0.61
$X_{6}\;\left(2013\right)$	0.57	0.58
$X_{7}\;\left(2014\right)$	0.61	0.63
$X_{8}\;\left(2015\right)$	0.57	0.59
$X_{9}\;\left(2016\right)$	0.63	0.65
$X_{10}\;\left(2017\right)$	0.54	0.56
$X_{11}\;\left(2018\right)$	0.58	0.60
$X_{12}\;\left(2019\right)$	0.54	0.56

**Table 3:** Point estimates of location.

Year	Mean $\left(mg/m^{3}\right)$	Median $M_{e}$ $\left(mg/m^{3}\right)$	Trimean TM $\left(mg/m^{3}\right)$	0.2-Trimmed Mean T(0.2) $\left(mg/m^{3}\right)$	0.3-Trimmed Mean T(0.3) $\left(mg/m^{3}\right)$	0.2-Winsorized Mean W(0.2) $\left(mg/m^{3}\right)$	0.3-Winsorized Mean W(0.3) $\left(mg/m^{3}\right)$	Andrew’s Wave $T_{wa}\left(2.4\pi \right)$ $\left(mg/m^{3}\right)$	Biweight $T_{bi}\left(9\right)$ $\left(mg/m^{3}\right)$
$2008\;\left(X_{1}\right)$	0.9859	0.8600	0.8950	0.9689	0.9388	0.9203	0.8885	0.9292	0.9261
$2009\;\left(X_{2}\right)$	0.9468	0.8300	0.8600	0.8905	0.8650	0.8835	0.8540	0.8878	0.8859
$2010\;\left(X_{3}\right)$	0.9494	0.8600	0.8800	0.9719	0.9461	0.8974	0.8760	0.8991	0.8988
$2011\;\left(X_{4}\right)$	0.8126	0.7200	0.7450	0.7923	0.7348	0.7648	0.7377	0.7708	0.7686
$2012\;\left(X_{5}\right)$	0.6881	0.6000	0.6250	0.6534	0.6170	0.6401	0.6228	0.6465	0.6446
$2013\;\left(X_{6}\right)$	0.6589	0.5800	0.6000	0.6565	0.6314	0.6118	0.5970	0.6150	0.6142
$2014\;\left(X_{7}\right)$	0.7038	0.6200	0.6400	0.6934	0.6716	0.6566	0.6345	0.6601	0.6590
$2015\;\left(X_{8}\right)$	0.6667	0.5800	0.5975	0.6280	0.5786	0.6135	0.5943	0.6120	0.6122
$2016\;\left(X_{9}\right)$	0.7317	0.6400	0.6650	0.7199	0.6445	0.6800	0.6623	0.6836	0.6822
$2017\;\left(X_{10}\right)$	0.6352	0.5500	0.5725	0.6212	0.6111	0.5854	0.5705	0.5921	0.5909
$2018\;\left(X_{11}\right)$	0.6600	0.5900	0.6075	0.6714	0.6789	0.6204	0.6035	0.6228	0.6221
$2019\;\left(X_{12}\right)$	0.6374	0.5500	0.5750	0.6077	0.5318	0.5932	0.5711	0.5925	0.5907

**Table 4:** Point estimates of scale.

Year	$s_{x}$ $\left(mg/m^{3}\right)$	$MAD_{mean}$ $\left(mg/m^{3}\right)$	MAD $\left(mg/m^{3}\right)$	SRH $\left(mg/m^{3}\right)$	LMS $\left(mg/m^{3}\right)$	$s^{W}\left(0.2\right)$ $\left(mg/m^{3}\right)$	$s_{wa}\left(2.4\pi \right)$ $\left(mg/m^{3}\right)$	$s_{bi}\left(9\right)$ $\left(mg/m^{3}\right)$	$C_{n}^{0.2}$ $\left(mg/m^{3}\right)$
$2008\;\left(X_{1}\right)$	0.5640	0.4339	0.3200	0.3400	0.2950	0.3309	0.5089	0.5137	0.4555
$2009\;\left(X_{2}\right)$	0.5297	0.4043	0.2900	0.3200	0.2700	0.3083	0.4660	0.4711	0.4191
$2010\;\left(X_{3}\right)$	0.4709	0.3548	0.2600	0.2700	0.2450	0.2658	0.4102	0.4152	0.3826
$2011\;\left(X_{4}\right)$	0.4358	0.3362	0.2500	0.2700	0.2350	0.2602	0.3940	0.3970	0.3644
$2012\;\left(X_{5}\right)$	0.3622	0.2750	0.2000	0.2100	0.1800	0.2027	0.3141	0.3181	0.2733
$2013\;\left(X_{6}\right)$	0.3620	0.2702	0.1900	0.2100	0.1750	0.1957	0.3047	0.3094	0.2733
$2014\;\left(X_{7}\right)$	0.3668	0.2746	0.2000	0.2100	0.1800	0.2025	0.3093	0.3141	0.2733
$2015\;\left(X_{8}\right)$	0.3661	0.2697	0.1800	0.1950	0.1700	0.1903	0.2925	0.2995	0.2551
$2016\;\left(X_{9}\right)$	0.4106	0.3072	0.2200	0.2400	0.2050	0.2255	0.3486	0.3532	0.3098
$2017\;\left(X_{10}\right)$	0.3945	0.2982	0.2200	0.2350	0.1950	0.2219	0.3416	0.3461	0.2915
$2018\;\left(X_{11}\right)$	0.3620	0.2744	0.2000	0.2150	0.1900	0.2083	0.3214	0.3244	0.2915
$2019\;\left(X_{12}\right)$	0.3654	0.2772	0.1900	0.2100	0.1750	0.2033	0.3159	0.3204	0.2733

**Table 5:** 95% confidence intervals ($$CI_{0.95}$$) and confidence interval lengths: $\left(T\left(0.2\right),s^{W}\left(0.2\right)\right)$ and $\left(T_{bi}\left(9\right),s_{bi}\left(9\right)\right)$.

Variable	$CI_{95}$	Lower Limit	Upper Limit	Length
$X_{1}$	$\left(T\left(0.2\right),s^{W}\left(0.2\right)\right)$	0.9571	0.9806	0.0235
	$\left(T_{bi}\left(9\right),s_{bi}\left(9\right)\right)$	0.9151	0.9370	0.0219
$X_{2}$	$\left(T\left(0.2\right),s^{W}\left(0.2\right)\right)$	0.8795	0.9015	0.0220
	$\left(T_{bi}\left(9\right),s_{bi}\left(9\right)\right)$	0.8758	0.8960	0.0202
$X_{3}$	$\left(T\left(0.2\right),s^{W}\left(0.2\right)\right)$	0.9625	0.9813	0.0188
	$\left(T_{bi}\left(9\right),s_{bi}\left(9\right)\right)$	0.8900	0.9076	0.0177
$X_{4}$	$\left(T\left(0.2\right),s^{W}\left(0.2\right)\right)$	0.7830	0.8016	0.0186
	$\left(T_{bi}\left(9\right),s_{bi}\left(9\right)\right)$	0.7601	0.7771	0.0170
$X_{5}$	$\left(T\left(0.2\right),s^{W}\left(0.2\right)\right)$	0.6462	0.6605	0.0144
	$\left(T_{bi}\left(9\right),s_{bi}\left(9\right)\right)$	0.6378	0.6513	0.0135
$X_{6}$	$\left(T\left(0.2\right),s^{W}\left(0.2\right)\right)$	0.6495	0.6635	0.0141
	$\left(T_{bi}\left(9\right),s_{bi}\left(9\right)\right)$	0.6075	0.6209	0.0133
$X_{7}$	$\left(T\left(0.2\right),s^{W}\left(0.2\right)\right)$	0.6863	0.7006	0.0144
	$\left(T_{bi}\left(9\right),s_{bi}\left(9\right)\right)$	0.6523	0.6657	0.0134
$X_{8}$	$\left(T\left(0.2\right),s^{W}\left(0.2\right)\right)$	0.6212	0.6347	0.0135
	$\left(T_{bi}\left(9\right),s_{bi}\left(9\right)\right)$	0.6058	0.6186	0.0128
$X_{9}$	$\left(T\left(0.2\right),s^{W}\left(0.2\right)\right)$	0.7119	0.7279	0.0160
	$\left(T_{bi}\left(9\right),s_{bi}\left(9\right)\right)$	0.6746	0.6897	0.0151
$X_{10}$	$\left(T\left(0.2\right),s^{W}\left(0.2\right)\right)$	0.6133	0.6291	0.0158
	$\left(T_{bi}\left(9\right),s_{bi}\left(9\right)\right)$	0.5835	0.5983	0.0148
$X_{11}$	$\left(T\left(0.2\right),s^{W}\left(0.2\right)\right)$	0.6640	0.6789	0.0149
	$\left(T_{bi}\left(9\right),s_{bi}\left(9\right)\right)$	0.6151	0.6290	0.0139
$X_{12}$	$\left(T\left(0.2\right),s^{W}\left(0.2\right)\right)$	0.6005	0.6149	0.0144
	$\left(T_{bi}\left(9\right),s_{bi}\left(9\right)\right)$	0.5838	0.5975	0.0137
